# Conjunctival inclusion cysts following small incision cataract surgery

**DOI:** 10.4103/0301-4738.67067

**Published:** 2010

**Authors:** Shylaja Narayanappa, S Dayananda, M Dakshayini, Suresh Babu Gangasagara, Venkatesh C Prabhakaran

**Affiliations:** Minto Ophthalmic Hospital, Bangalore, India; 1 Department of Pathology, Bangalore Medical College & Research Institute (BMC & RI), Bangalore, India

**Keywords:** Conjunctival inclusion cyst, manual small incision cataract surgery

## Abstract

The occurrence of acquired conjunctival inclusion cysts following various ophthalmic surgeries such as strabismus surgery, scleral buckling, pars plana vitrectomy, ptosis surgery and phacoemulsification has been reported. We report two cases of conjunctival inclusion cysts following manual Small Incision Cataract Surgery (SICS) in two male patients aged 65 and 67 years. The cysts originated from the scleral tunnel used for manual SICS. Both were treated by excision and confirmed histopathologically. No recurrence was noted at three months follow-up. To our knowledge, conjunctival inclusion cysts following SICS have not been reported previously. Careful reflection of conjunctiva during tunnel construction and posterior chamber intraocular lens implantation may prevent their occurrence.

Conjunctival inclusion cysts are benign cysts filled with clear serous fluid containing shed cells or gelatinous mucous material.[1] They can be congenital or acquired.[2] Acquired cysts occur following traumatic or surgical implantation of conjunctival epithelium, and are much more common.[3] We report two cases of conjunctival inclusion cysts following manual small incision cataract surgery (SICS). To our knowledge, this complication of manual SICS has not been reported previously.

## Case Reports

### Case 1

A 65-year-old man came with complaints of a swelling in the left eye noticed since one month. It was painless and gradually increasing in size. He had undergone manual SICS with posterior chamber intraocular lens (PCIOL) implantation in Left Eye (LE) two years ago. On examination both eyes had best corrected visual acuity (BCVA) of 20/40 and were pseudophakic with PCIOLs. A solitary cystic swelling was present at the superior limbus in the LE, overlying the previous scleral tunnel incision, measuring 8 X 8 mm and containing clear fluid [Fig. 1A]. The remainder of the ophthalmic examination was unremarkable. He was posted for surgical excision of the cyst under local anesthesia. The conjunctiva around the cyst was separated by blunt dissection, the cyst ruptured while excising it from the base. The sclera underneath was cauterized. The conjunctiva was re-approximated to the limbus. The excised cyst was subjected to histopathological examination, which revealed a cyst lined by non-keratinized stratified squamous epithelium suggestive of a conjunctival inclusion cyst [Fig. 1B]. He has been followed up for three months, without any signs of recurrence.

**Figure 1A:**
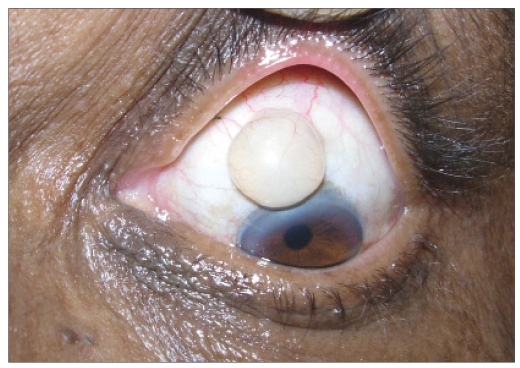
Preoperative photograph of Case 1, showing a conjunctival inclusion cyst at superior limbus containing clear fluid

**Figure 1B:**
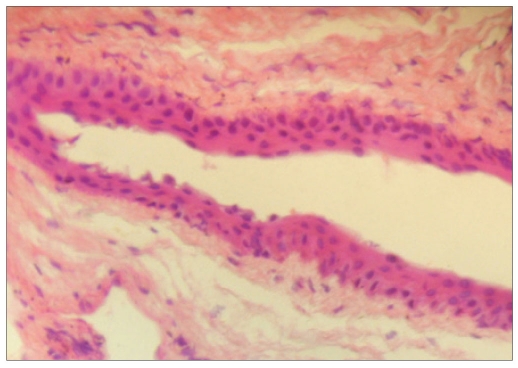
Photomicrograph of the cyst lined by non-keratinized stratified squamous epithelium (H&E, ×100)

### Case 2

A 67-year-old man came with complaint of a swelling in his Right Eye (RE) noticed since two months, initially smaller in size, gradually increased to present size and associated with foreign body sensation. He had undergone manual SICS with PCIOL implantation in RE eight months ago. On examination, RE had a BCVA of 20/60, and was pseudophakic with PCIOL. A solitary oval cystic swelling was present at the superior limbus in RE, measuring 11 mm × 9 mm, and containing clear fluid [Fig. 2A]. The LE had a BCVA of counting fingers 4 meters, with posterior subcapsular cataract. The anterior segment and fundus examination were unremarkable. He was posted for surgical excision of the cyst under topical anesthesia. The conjunctiva overlying the cyst was separated by blunt dissection, the cyst was excised at the base, but as with the previous case it ruptured during excision. The underlying sclera was cauterized. The conjunctiva was re-approximated to the limbus by a subconjunctival injection of 0.5 ml gentamicin. The excised cyst was subjected to histopathological examination, which revealed a cyst lined by non-keratinized stratified squamous epithelium showing occasional apical snouts [Fig. 2B], again suggestive of conjunctival inclusion cyst. He also has been followed up for three months without any signs of recurrence.

**Figure 2A:**
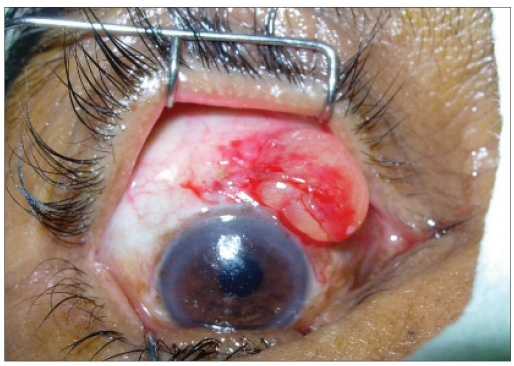
Intra-operative clinical photograph of Case 2, showing an oval conjunctival cyst at superior limbus; the overlying conjunctiva has been reflected

**Figure 2B:**
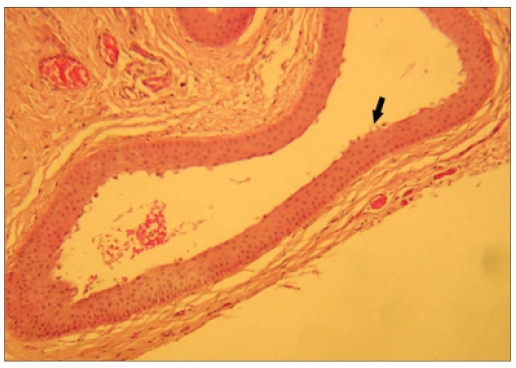
Photomicrograph of the cyst, showing non-keratinized stratified squamous epithelium lining with occasional apical snouts (arrow) (H&E, ×100)

## Discussion

Conjunctival inclusion cysts can be congenital or acquired. Acquired conjunctival inclusion cysts occur following trauma or surgery. They are most common following strabismus surgery,[3] but may also occur following other ophthalmic surgeries such as pars plana vitrectomy,[4] scleral buckling,[5] Ahmed glaucoma valve insertion,[6] and ptosis surgery.[7] While conjunctival inclusion cyst following phacoemulsification surgery has been reported,[8] there are no reports following manual SICS. These cysts are generally asymptomatic, or may cause a mild foreign body sensation or cosmetic concerns. The chief differential diagnosis is a filtering bleb. Possible complications are infection and the rare possibility of epithelial ingrowth through the tunnel wound. These cysts may disappear spontaneously; however, persistent cases require treatment. Surgical excision of the cyst is the best treatment. Thermal cautery under slit-lamp visualization[9] or YAG laser of the cyst can also be performed.[10]

From our cases, we note that conjunctival inclusion cysts can occur following manual SICS. The conjuntival tissue may be implanted during the construction of the tunnel wound or due to dragging of conjunctiva during PCIOL implantation. This complication may be prevented by paying careful attention to reflection of the conjunctiva prior to scleral wound construction and by avoiding contact between the IOL and conjunctiva during intraocular lens implantation.
